# Psychotherapie nach einem Suizidversuch – Evidenzlage und Bewertung

**DOI:** 10.1007/s00103-021-03466-y

**Published:** 2021-12-08

**Authors:** Tobias Teismann, Anja Gysin-Maillart

**Affiliations:** 1 Forschungs- und Behandlungszentrum für Psychische Gesundheit, Fakultät für Psychologie, Ruhr-Universität Bochum, Massenbergstr. 11, 44788 Bochum, Deutschland; 2 Translationales Forschungszentrum, Universitätsklinik für Psychiatrie und Psychotherapie Bern, Universität Bern, Bern, Schweiz; 3 Abteilung für Medizinische Psychologie und Medizinische Soziologie, Universität Leipzig, Leipzig, Deutschland; 4 Abteilung klinische Suizidforschung, Departement für klinische Forschung und Psychiatrie, Medizinische Fakultät, Universität Lund, Lund, Schweden

**Keywords:** Suizidversuch, Suizid, Suizidprävention, Metaanalysen, Kognitive Verhaltenstherapie, Suicide attempt, Suicide, Suicide prevention, Meta-analyses, Cognitive-behavioural therapy

## Abstract

Suizidversuche gelten als einer der wichtigsten Risikofaktoren für Suizide. Vor diesem Hintergrund wurden in den letzten Jahren diverse Psychotherapieangebote für Personen nach einem Suizidversuch entwickelt und untersucht. Im Rahmen dieses Artikels wird der aktuelle Stand der Effektivitätsforschung zusammengefasst, es werden Beispiele für erfolgreiche suizidfokussierte Psychotherapieprogramme gegeben und der gegenwärtige Forschungs- und Wissensstand wird kritisch reflektiert. Die Ergebnisse von 2 aktuellen Cochrane-Reviews zur Psychotherapie nach selbstverletzendem Verhalten im Kindes‑, Jugend- und Erwachsenenalter sowie Befunde aus 14 weiteren Metaanalysen zur psychologischen Suizidprävention, die in den vergangenen 5 Jahren publiziert wurden, werden überblicksartig dargestellt.

Die kognitive Verhaltenstherapie (KVT) und die dialektisch-behaviorale Therapie (DBT) haben sich als effektiv erwiesen. Insgesamt sind die gemittelten Effektstärken jedoch von geringer Größe und diverse methodische Probleme verunmöglichen weitreichende Schlussfolgerungen. Grundsätzlich kommt der suizidspezifischen Psychotherapie in der individuumszentrierten Suizidprävention besondere Bedeutung zu; die empirische Fundierung und Dissemination entsprechender Programme sind jedoch noch unzureichend.

## Abstract (en)

Suicide attempts are considered one of the most important risk factors for suicide. Based on this, various psychotherapeutic treatments for persons after a suicide attempt have been developed and evaluated in recent years. In this article, the current state of efficacy research is outlined, examples of successful suicide-focused psychotherapies are presented, and the current state of research and knowledge is critically reflected upon. The results of two recent Cochrane reviews of psychotherapy following self-injurious behavior in childhood, adolescence, and adulthood, as well as findings from 14 other meta-analyses of psychological suicide prevention published in the past five years, are presented.

Cognitive behavioral therapy and dialectical behavioral therapy have been shown to be effective. Overall, however, the averaged effect sizes are of small magnitude and various methodological problems make it impossible to draw far-reaching conclusions. In principle, suicide-specific psychotherapy is of particular importance in individual-centered suicide prevention; however, the empirical foundation and dissemination of appropriate programs are still insufficient.

## Hintergrund

Im Jahr 2020 starben in der Bundesrepublik Deutschland 9206 Menschen durch einen Suizid [1]. Die Rate von Suizidversuchen liegt naturgemäß um ein Vielfaches höher. So wird die Lebenszeitprävalenz von Suizidversuchen in Deutschland mit 1,7 % bei Erwachsenen [2] und mit 9 % bei Jugendlichen [3] beziffert. Suizidversuche gelten – neben psychischen Erkrankungen – als zentraler Risikofaktor für Suizide [4]. In einer Metaanalyse kommen Caroll et al. [5] zu dem Ergebnis, dass das Risiko, durch einen Suizid zu versterben, im ersten Jahr nach einem Suizidversuch bei 1,6 %, nach 5 Jahren bei 3,9 % und nach 10 Jahren bei 4,2 % liegt. Das Risiko eines erneuten Suizidversuchs wird im ersten Jahr auf 16,3 % und binnen der ersten 5 Folgejahre auf 22,4 % geschätzt [5]. Bei den allermeisten Betroffenen bleibt ein Suizidversuch somit ein singuläres Ereignis in ihrem Leben; gleichzeitig ist das Risiko, an einem Suizid zu sterben, nie so hoch wie im ersten Jahr nach einem Suizidversuch. Entsprechend stellt sich die Frage, mit welchen psychotherapeutischen Maßnahmen dem erhöhten Wiederholungs- und Mortalitätsrisiko begegnet werden kann.

Ziel dieser Arbeit ist es, einen narrativen Überblick über Psychotherapieverfahren zu geben, die speziell zur Behandlung von Personen nach Suizidversuch entwickelt und evaluiert wurden. Hierzu werden Befunde aus Metaanalysen – insbesondere 2 aktuelle Cochrane-Reviews – zusammengefasst und Beispiele für effektive Behandlungsansätze gegeben. Es folgt eine kritische Würdigung der gegenwärtigen Befundlage.

## Evidenzen aus systematischen Reviews und Metaanalysen

Im Rahmen von 2 aktuellen Cochrane-Reviews haben Witt et al. [6, 7] Studien zur Effektivität psychosozialer Interventionen bei der Behandlung von Erwachsenen und von Kindern/Jugendlichen zusammengefasst. Einbezogen in die Metaanalysen wurden ausschließlich randomisiert kontrollierte Studien, in denen eine psychosoziale Intervention mit einer Vergleichsbedingung (zumeist Regelversorgung, engl. „treatment as usual“, TAU) verglichen wurde. Ein weiteres Einschlusskriterium sah vor, dass sich alle Patient*innen innerhalb der letzten 6 Monate vor Studieneinschluss absichtsvoll selbstverletzt (engl. „self-harm“) haben mussten. Primäres Outcome-Maß war das erneute Auftreten intentionaler Selbstverletzung. Insgesamt wurden 76 Studien (*N* = 21.414) mit Fokus auf erwachsene Patient*innen [6] und 17 Studien (*N* = 2280) mit Fokus auf Kinder und Jugendliche [7] identifiziert.

Bei einer weitgehend unzureichenden Studiengüte fanden sich bei Erwachsenen Hinweise darauf, dass kognitive Verhaltenstherapie (KVT) 6 (OR 0,52, 95 % Konfidenzintervall (KI) 0,38–0,70; *n* = 1260; Anzahl der Studien (*k)* = 12) und 12 Monate (OR 0,81, 95 % KI 0,66–0,99; *n* = 2458; *k* = 9) nach Therapieende zu einer Reduktion erneuter Selbstverletzungen beiträgt [6]. Zudem zeigte sich eine Reduktion von Suizidgedanken zum Therapieende und 6 Monate nach Therapieende (siehe auch [8]). In Bezug auf die dialektisch-behaviorale Therapie (DBT) fanden Witt et al. [6] keinen Effekt im Hinblick auf das Auftreten von selbstverletzendem Verhalten, wohl aber mit Blick auf die Häufigkeit, mit der erwachsene Patient*innen selbstverletzendes Verhalten zu Therapieende zeigten (Mean Difference (MD) −5,00, 95 % KI −8,92 bis −1,08; *n* = 659; *k* = 7). In Bezug auf unterschiedliche DBT-Gestaltungsformen fanden sich keine differenziellen Befunde und es zeigte sich kein Behandlungseffekt hinsichtlich Suizidgedanken. In weiteren Studien erwiesen sich die mentalisierungsbasierte Therapie (MBT) und eine auf Emotionsregulation fokussierende Gruppentherapie als effektiv hinsichtlich einer Reduktion selbstverletzenden Verhaltens.

Im Kindes- und Jugendalter zeigten sich positive Effekte ausschließlich für die dialektisch-behaviorale Therapie (DBT-A) zum Postzeitpunkt (OR 0,46, 95 % KI 0,26–0,82; *n* = 270; *k* = 4); weder in Bezug auf KVT noch MBT fanden sich entsprechende Wirksamkeitsnachweise [7].

Kritisch lässt sich anmerken, dass in beiden Cochrane-Reviews – im Sinne der englischen Tradition [9] – nicht zwischen Selbstverletzungen in suizidaler und nichtsuizidaler Absicht differenziert wird. Es bleibt daher unklar, ob und inwieweit die Befunde gleichermaßen für nichtsuizidale Selbstverletzungen wie für Suizidversuche gelten. Vor diesem Hintergrund wurde von Gøtzsche und Gøtzsche [10] eine Reanalyse auf Basis einer älteren Fassung des Cochrane-Reviews [11] zur psychosozialen Suizidprävention im Erwachsenenalter vorgenommen. In die Metaanalyse wurden 10 randomisiert kontrollierte Studien eingeschlossen, in denen kognitive Verhaltenstherapie mit TAU verglichen wurde. Primäres Outcome-Maß waren erneute Suizidversuche (im Unterschied zu einem integrierten Maß „self-harm“). Es zeigte sich eine nahezu 50 %ige Reduktion des Risikos eines erneuten Suizidversuchs infolge einer KVT im Vergleich zu einer TAU-Behandlung (Risk Ratio 0,47, 95 %, KI 0,30–0,73; *N* = 1241). Die Befunde verweisen zum einen auf die Effektivität (einzelner) KVT-Behandlungsprogramme (siehe unten) und zum anderen auf die Bedeutung, zwischen suizidaler und nichtsuizidaler Selbstverletzung als Outcome-Maß zu differenzieren.

In weiteren Metaanalysen wurde der Effekt psychotherapeutischer Suizidprävention untersucht, ohne dass vorangegangenes selbstverletzendes Verhalten als Einschlusskriterium gefordert wurde. Tab. 1 gibt einen Überblick über entsprechende Metaanalysen, die in den Jahren 2016–2021 publiziert wurden. Dabei wurden Metaanalysen, die unkontrollierte Prä-Post-Designs [12–14] inkludieren, genauso wenig berücksichtigt wie eine Metaanalyse zu Kurz- und Kontaktinterventionen [15], eine Metaanalyse zum Vergleich verschiedener TAU-Bedingungen [16] und eine Metaanalyse zu Behandlungsabbrüchen [17].

**Table 1:** 

Autor*innen	Jahr	Therapiemethode im Fokus	Anzahl Studien (*k*)	Anzahl Proband*innen (*N*)	Primäres Outcome-Maß	Ergebnis
Bornheimer et al. [18]	2020	Psychosoziale Interventionen bei Psychosen	11	4829	Suizidgedanken, Suizidversuch, Suizid	BG > KG bzgl. Suizidgedanken (OR = 0,73, 95 % KI, 0,55–0,97) BG > KG bzgl. Suizide (OR = 0,45, 95 % KI 0,30–0,68) BG = KG bzgl. Suizidversuche
Briggs et al. [19]	2019	Psychoanalyse/Psychodynamische Therapie (PA/PD)	12	999	Suizidversuch, „self-harm“	PA/PD > KG bzgl. Suizidversuche (OR = 0,47, 95 % KI 0,27–0,80) PA/PD > KG bzgl. „self-harm“ (6 Monate FU: OR = 0,27, 95 % KI 0,10–0,66) PA/PD = KG bzgl. Anzahl Suizidversuche, Anzahl Self-harm-Episoden, „self-harm“ (12 Monate FU)
Büscher et al. [20]	2020	Internetbasierte kognitive Verhaltenstherapie (KVT)	6	1567	Suizidgedanken	KVT > KG bzgl. Suizidgedanken (SMD = −0,29; 95 % KI −0,40 bis −0,19)
Calati und Courtet [21]	2016	Psychotherapie	32	4114	Suizidversuch, NSSI	BG > KG bzgl. Suizidversuche (Suizidversuchsrate: 9,12 % vs. 15,71 %) BG = KG bzgl. NSSI
DeCou et al. [22]	2019	Dialektisch-behaviorale Therapie (DBT)	18	987	Self-harm	DBT > KG bzgl. „self-harm“ (*d* = −0,32, 95 % KI −0,47 bis −0,17) DBT = KG bzgl. Suizidgedanken
Fox et al. [23]	2020	Psychosoziale Interventionen, Psychotherapie, Pharmakotherapie	1125	–	Suizidgedanken, Suizidversuche, „self-harm“, NSSI, Suizid	BG > KG bzgl. Suizidgedanken (*g* = −0,09; 95 % KI −0,15 bis −0,02) BG = KG bzgl. Suizidversuche, Suizid, NSSI, „self-harm“ (jeweils Posttreatment)
Kothgassner et al. [24]	2020	Psychotherapie bei Jugendlichen	25	2962	Self-harm, Suizidgedanken	BG > KG bzgl. „self-harm“ (*d* = 0,13, 95 % KI 0,04–0,22) BG > KG bzgl. Suizidgedanken (*d* = 0,31, 95 % KI 0,12–0,50)
Leavey und Hawkins [25]	2017	Kognitive Verhaltenstherapie	26	2790	Suizidalität (Suizidgedanken, Suizidversuche)	BG (Face-to-Face-Behandlung) > KG bzgl. Suizidalität (SMD = −0,24, 95 % KI −0,41 bis −0,07)
Meerwjik et al. [26]	2016	Spezifische vs. unspezifische psychosoziale und psychotherapeutische Interventionen	44	13.369 (Postmessung) 11.887 (FU-Messung)	Suizidversuche	Direkte Intervention = indirekte Intervention zum Postzeitpunkt (OR = 0,62 vs. 0,93, *t* = −2,04, *p* = 0,06; *d* = 0,77) Direkte Intervention = indirekte Intervention zum FU-Zeitpunkt (OR = 0,65 vs. 0,82, *t* = −1,20, *p* = 0,25; *d* = 0,47)
Padmanathan et al. [27]	2020	Psychosoziale Interventionen/Pharmakotherapie bei Substanzkonsumstörungen	6	468	Suizidalität (Suizidgedanken, „self-harm“)	BG > KG bzgl. Suizidalität (*d* = −0,20, 95 % KI −0,39 bis −0,00)
Riblet et al. [28]	2017	Psychosoziale Interventionen, Psychotherapie, Pharmakotherapie	24	3056	Suizid	KVT = KG bzgl. Suizid (OR = 0,34, 95 % KI 0,12–1,03)
Swift et al. [29]	2021	Collaborative Assessment and Management of Suicidality (CAMS)	9	749	Suizidgedanken	CAMS > KG bzgl. Suizidgedanken (*d* = 0,25, 95 % KI 0,07–0,42) CAMS = KG bzgl. Suizidversuche, „self-harm“
Torok et al. [30]	2020	Internetbasierte Therapie	16	4398	Suizidgedanken	BG > KG bzgl. Suizidgedanken (Hedges *g* = −0,18, 95 % KI −0,27 bis −0,10)

*BG* Behandlungsgruppe, *FU* Follow-up-Erhebung (Nachuntersuchung), *KG* Kontrollbedingung, *KI* Konfidenzintervall, *NSSI* Nonsuicidal Self-injury (dt. nichtsuizidale Selbstverletzung), *OR* Odds Ratio, *SMD* standardisierte Mittelwertdifferenz

Alles in allem findet sich in Metaanalysen – mit variierenden Einschlusskriterien – ein präventiver Effekt psychotherapeutischer Interventionen auf die Rate von Patient*innen, die erneute Suizidversuche und/oder Selbstverletzungen vornehmen, sowie auf die Reduktion von Suizidgedanken. Ein präventiver Effekt auf die Anzahl an Suiziden findet sich nur in einer einzelnen Studie [27]. Grundsätzlich scheinen Verfahren, die suizidales Erleben und Verhalten zum expliziten Fokus der Behandlung machen, effektiver zu sein als Verfahren, die auf die Reduktion assoziierter Psychopathologie (z. B. Depression, Hoffnungslosigkeit) abzielen [26, 30], allerdings braucht es diesbezüglich noch weitere Bestätigung [23]. Einschränkend ist schlussendlich hervorzuheben, dass (1) die Effekte zumeist nur von geringer Größe sind, (2) sich – in Abhängigkeit von den Einschlusskriterien, Outcome-Variablen und Messzeitpunkt(en) – nicht in allen Metaanalysen präventive Effekte finden, (3) insbesondere Onlineangebote mit einer hohen Abbrecherquote konfrontiert sind, (4) für Kinder und Jugendliche nur wenige effektive Behandlungsangebote bereitstehen, (5) nicht in allen Studien zwischen Suizidversuchen und nichtsuizidaler Selbstverletzung differenziert wird und (6) die methodische Qualität eines Großteils der Studien gering ist (57,58 %; [23]).

Insgesamt würde das Feld davon profitieren, wenn systematisch solche Therapieansätze in methodisch adäquaten (Replikations‑)Studien überprüft werden würden, die sich in ersten Untersuchungen als besonders effektiv herausgestellt haben, die potenziell störungs- und altersübergreifend einsetzbar sowie in manualisierter Form (Einzelschritte der Therapie in einem Handbuch dargelegt) verfügbar sind.

## Beispiele für effektive Psychotherapieprogramme

Vor diesem Hintergrund sollen im Folgenden verschiedene Formen der KVT skizziert werden: die kognitive Therapie für suizidale Patient*innen (KT-SP; [31]), die kurze kognitive Verhaltenstherapie zur Suizidprävention (KVT-SP; [32]) und das Attempted Suicide Short Intervention Program (ASSIP; [33]). Sämtliche dieser Behandlungsprogramme haben sich in ersten Studien als effektiv erwiesen [10] und entsprechen den oben genannten Kriterien.

### Kognitive Therapie und kurze kognitive Verhaltenstherapie suizidaler Patienten

Die kognitive Therapie suizidaler Patienten (KT-SP) und die kurze kognitive Verhaltenstherapie zur Suizidprävention (KVT-SP) verstehen sich als Kurzzeittherapien (10 bis 12 Sitzungen), die in Ergänzung zu anderen Therapien eingesetzt werden können und die sich in 3 Therapiephasen untergliedern lassen: die Eingangsphase, die mittlere Therapiephase und die Abschlussphase.

In der *Eingangsphase* geht es darum, die suizidale Krise zu verstehen, Ansatzpunkte für die Behandlung zu identifizieren, eine Risikoabschätzung vorzunehmen und krisentherapeutische Strategien zu implementieren. Die Behandlung nimmt ihren Ausgangspunkt mit dem sogenannten narrativen Interview, bei dem Betroffene aufgefordert werden, die Geschehnisse, die zum Suizidversuch geführt haben, in ihren Worten zu erzählen (vgl. ASSIP Punkt 1, Tab. 2). In einer sich anschließenden hochauflösenden Kognitionsanalyse werden die Abläufe unmittelbar um den Suizidversuch herum dann nochmals detailliert betrachtet. Ergänzend wird in der Eingangsphase eine Risikoabschätzung vorgenommen, es wird ein Notfallplan erstellt und es werden Gründe, die für das Weiterleben sprechen, herausgearbeitet und verschriftlicht. Zusätzlich wird eine sogenannte Hope Box erstellt, d. h., die Patienten werden darin unterstützt, eine Schachtel mit Gegenständen zu füllen, die an die persönlichen Gründe weiterzuleben erinnern.

In der mittleren *Therapiephase* werden – auf der Basis der individuellen Fallkonzeption – solche KVT-Standardmethoden gewählt, die im individuellen Fall das größte Potenzial haben, einem weiteren Suizidversuch vorzubeugen. Im Einzelnen kann es also darum gehen, dass (1) kognitive Infragestellungsmethoden genutzt werden, um suizidbezogene Annahmen/Vorstellungen („Ich bin eine Last für andere“) zu modifizieren, (2) Problemlösetrainings verwendet werden, um dem Eindruck der Ausweglosigkeit entgegenzutreten, (3) soziale Ängste und Inkompetenzen angegangen werden, um die soziale Integration zu fördern, (4) Skills und Achtsamkeitsübungen vermittelt werden, um den Umgang mit emotionalem Schmerz zu erleichtern, und (5) Maßnahmen zur Verbesserung des Schlafs und der Entspannungsfähigkeit genutzt werden, um einer weiteren emotionalen Eskalation entgegenzuwirken. Alle diese Techniken und Strategien werden in gleicher oder vergleichbarer Form auch in der kognitiven Verhaltenstherapie nichtsuizidaler Patient*innen eingesetzt – sie sind also wenig spezifisch. Der Unterschied in der Anwendung ergibt sich ausschließlich dadurch, dass die Methoden, ausgehend von einem genauen Verständnis, wie eine suizidale Krise bei einem Betroffenen „funktioniert“, passgenau darauf abzielen, erneuten suizidalen Krisen, Suizidversuchen und Suiziden vorzubeugen.

In der *Abschlussphase* wird eine sogenannte Rückfallpräventionsübung (Relapse Prevention Task) genutzt, um die erworbenen Fertigkeiten imaginativ zu erproben. Bei der Rückfallpräventionsintervention handelt es sich um eine Imaginationsübung, in der Patient*innen angeleitet werden, sich eine zurückliegende und eine antizipierte zukünftige suizidale Krise imaginativ zu vergegenwärtigen und den Einsatz von Bewältigungsstrategien in sensu (in der Vorstellung) zu erproben. Die Technik dient sowohl zur Konsolidierung der Therapieinhalte als auch zur Kontrolle, ob Patient*innen in der Lage sind, die entwickelten Strategien – zumindest in der Vorstellung – einzusetzen.

Die Effektivität des beschriebenen Vorgehens konnte in 2 unabhängigen Studien gezeigt werden: Brown et al. [34] verglichen die KT-SP mit einer TAU-Bedingung bei 120 Patient*innen, die kurz zuvor einen Suizidversuch unternommen hatten. Im Studienzeitraum von 18 Monaten wiesen die Patient*innen der Behandlungsgruppe eine nahezu 50 % niedrigere Suizidversuchsrate auf als die Patient*innen der TAU-Bedingung (24 % KT vs. 42 % TAU). In der Untersuchung von Rudd et al. [35] erhielten 152 suizidale Soldat*innen entweder die KVT-SP oder eine TAU-Behandlung. Im Studienzeitraum von 24 Monaten wiesen die Patient*innen der Behandlungsgruppe eine nahezu 60 % niedrigere Suizidversuchsrate auf als die Patient*innen der TAU-Bedingung (14 % KVT vs. 40 % TAU). Die Befunde unterstreichen sehr deutlich das Potenzial des Behandlungsansatzes. Spezifikationen des allgemeinen therapeutischen Vorgehens liegen für Jugendliche und alte Menschen [31] sowie den stationären Kontext [36] vor. Eine Adaptation für den deutschen Sprachraum steht bislang aus.

### Attempted Suicide Short Intervention Program (ASSIP)

Eine kurztherapeutische Behandlungsalternative stellt das Attempted Suicide Short Intervention Program (ASSIP) von Gysin-Maillart [33] dar. Die Behandlung umfasst insgesamt 3–4 Sitzungen mit einem anhaltenden Kontaktangebot durch das Zusenden von Briefen. Die Behandlung zielt darauf ab, die Hintergründe einer suizidalen Krise zu klären und Strategien zur Prävention erneuter suizidaler Handlungen zu entwickeln. ASSIP wird dabei immer als Zusatzangebot zu einer regulär laufenden Behandlung (ambulant, teilstationär, stationär) angeboten (Tab. 2).

**Table 2:** 

ASSIP-Sitzungsinhalte	
(1)	In der *ersten Sitzung*, dem narrativen Interview, werden die Patient*innen gebeten, ihre Geschichte, die zum Suizidversuch geführt hat, zu erzählen („Ich möchte Sie bitten mir zu erzählen, wie es zu diesem Suizidversuch kam“). Den Patient*innen wird Raum gegeben, die Geschichte so zu erzählen, wie sie sie erlebt haben. Therapeut*innen sind angehalten, Pausen zuzulassen und die Erzählung nicht durch Fragen zu unterbrechen. Erst wenn Patient*innen mit ihrer Geschichte zum Ende kommen, sollten Therapeut*innen offene Verständnisfragen klären. Das Ziel des narrativen Interviews besteht darin, ein gemeinsames, patientenorientiertes Verständnis der suizidalen Krise in einem biografischen Kontext zu entwickeln. Im Mittelpunkt stehen die narrative Kompetenz von Patient*innen und der Aufbau einer frühen therapeutischen Beziehung. Die Sitzung wird auf Video aufgezeichnet. Psychoedukative Unterlagen („Suizid ist keine überlegte Handlung“) werden den Patient*innen mit nach Hause gegeben
(2)	In der *zweiten Sitzung* wird der Hausaufgabentext besprochen und persönliche Erfahrungen mit den wichtigen theoretischen Konzepten in Zusammenhang gebracht. Im Anschluss wird ein Video-Playback durchgeführt, in dem Patient*in und Therapeut*in Seite an Seite sitzen und ausgewählte Sequenzen des aufgezeichneten Narrativs anschauen. Dabei werden individuelle Muster, die zu suizidalem Verhalten führten, sowie persönliche Vulnerabilitäten und Trigger-Ereignisse identifiziert. Ziel des Video-Playbacks ist zudem, den mentalen Zustand (den sogenannten suizidalen Modus) der Patient*innen in einem sicheren Setting zu reaktivieren und gleichzeitig den Übergang des erlebten psychischen Schmerzes/Stresses hin zu suizidalem Verhalten Schritt für Schritt zu analysieren. Automatische Gedanken, Gefühle, physiologische Veränderungen und daraus resultierende Verhaltensmuster werden identifiziert und präventive Verhaltensmaßnahmen erarbeitet. Zugleich werden eine kognitive Umstrukturierung und eine emotionale Neuintegration ermöglicht. Für die dritte Sitzung bereiten die Therapeut*innen eine schriftliche Zusammenfassung der Abläufe der suizidalen Krise vor
(3)	In der *dritten Sitzung* wird dieser schriftliche Entwurf der individuellen und suizidspezifischen Fallkonzeption gemeinsam überarbeitet. Muster, Abläufe und längerfristige Therapieziele, die im Zusammenhang mit suizidalen Krisen stehen, aber auch persönliche Warnzeichen und suizidspezifische Bewältigungsstrategien werden erarbeitet und in der Fallkonzeption suizidalen Verhaltens festgehalten. Anschließend werden diese – im Sinne eines Notfallplans – auf einen kreditkartengroßen Leporello (Zick-Zack-Faltheft) kopiert. Zusätzlich erhalten die Patient*innen eine Notfallkarte mit wichtigen Notfallnummern, um einen einfachen Zugang zum Helfersystem zu garantieren
(4)	In der *vierten Sitzung*, die optional angeboten wird, werden die erarbeiteten Strategien im Rahmen einer erneuten Videoexposition eingeübt: Die Patient*innen betrachten die Videoaufnahme aus der ersten Sitzung und werden aufgefordert, die Geschichte ihres suizidalen Erlebens und Verhaltens mit den erarbeiteten Strategien probeweise zu unterbrechen. Im Anschluss an die Sitzungen bekommen die Patient*innen über 2 Jahre hinweg regelmäßig halbstandardisierte Briefe zugeschickt. Hierbei werden die Patient*innen daran erinnert, dass auch in Zukunft suizidale Krisen auftreten können und sie dabei ihre eigenen erarbeiteten Strategien nutzen sollen. Diese Vorgehensweise dient dem Ziel, eine lockere, aber verbindliche therapeutische Verankerung zu halten und einen einfachen Zugang zum Helfersystem zu gewährleisten

Die Effektivität von ASSIP wurde bislang in einer randomisiert kontrollierten Studie gezeigt: Gysin-Maillart und Kolleg*innen [37] verglichen ASSIP plus TAU mit einer reinen TAU-Bedingung bei 120 Patient*innen, die zuvor einen Suizidversuch unternommen hatten. Im Studienzeitraum von 24 Monaten kam es zu 5 Suizidversuchen in der ASSIP-Bedingung und zu 41 Suizidversuchen in der Kontrollbedingung. Der Anteil an Patient*innen, die einen oder mehrere Suizidversuche unternahmen, lag bei 8,3 % (*n* = 5) in der ASSIP-Bedingung, im Vergleich zu 26,7 % (*n* = 16) in der Kontrollbedingung. Patient*innen der ASSIP-Gruppe hatten ein annähernd 80 % geringeres Risiko, einen späteren Suizidversuch zu unternehmen, als Patient*innen der TAU-Bedingung.

Die Ergebnisse verweisen auf den präventiven Nutzen von ASSIP. Allerdings bleiben die Ergebnisse verschiedener – derzeit laufender – Replikationsstudien und die damit verbundenen klinischen Implikationen abzuwarten.

## Diskussion

Die beschriebenen Interventionsbeispiele verweisen auf die Möglichkeit effektiver psychotherapeutischer Suizidprävention und empfehlen sich für eine fortgesetzte empirische Überprüfung.

Gleichwohl behalten die im Rahmen metaanalytischer Untersuchungen sichtbar gewordenen Probleme ihre Gültigkeit: Die gemittelten Effektstärken sind gering (und dies in Bezug auf sämtliche suizidalitätsbezogene Outcome-Maße und im Vergleich zu TAU-Bedingungen)^1^, die methodische Qualität eines Großteils der Studien ist unzureichend und es fehlt nahezu vollständig an Replikationsstudien. Insbesondere Letzteres ist hierbei kritisch zu werten. So gibt es – vor allem aus dem Bereich der psychotherapeutischen Suizidprävention im Kindes- und Jugendalter – verschiedene Beispiele, in denen initial vielversprechende Behandlungsergebnisse [38–40] in Folgestudien nicht repliziert werden konnten [41–43].

Zudem wurden nur in Ausnahmefällen verschiedene aktive Behandlungsformen gegeneinander getestet, sodass sich zur Über- oder Unterlegenheit verschiedener Psychotherapieverfahren sowie zu differenziellen Indikationen bislang keine Aussagen treffen lassen. Es fehlt an Studien zur Effektivität suizidfokussierter Psychotherapieverfahren im Kontext unterschiedlicher Störungsbilder (u. a. Essstörungen), Settings (u. a. stationäre Psychotherapie) und im Rahmen unterschiedlicher Gesundheitssysteme (z. B. Deutschland, Schweiz, USA). Schlussendlich fehlt es an ausreichend gepowerten multizentrischen Studien, in denen die Prävention von Suiziden, anstatt der Prävention von Suizidversuchen und/oder Suizidgedanken als Outcome-Maß gewählt wurde.

Trotz aller berechtigten Kritik scheint die suizidfokussierte Psychotherapie die derzeit aussichtsreichste Kandidatin unter den individuumszentrierten Interventionen zu sein: Mit Ausnahme von Lithium [44] konnte bislang für kein Medikament eine stabile antisuizidale Wirkung nachgewiesen werden [23] und andere somatische Behandlungsverfahren (Elektrokrampftherapie, Schlafentzug) wurden zu selten untersucht, um eine Einschätzung vornehmen zu können. Vor dem Hintergrund der Ergebnisse der Cochrane-Reviews von Witt und Kolleg*innen [6, 7] empfehlen sich insbesondere KVT, DBT und MBT für weitere Wirksamkeitsstudien.

## Fazit

Es lässt sich festhalten, dass spezifische psychotherapeutische Angebote für Patient*innen nach einem Suizidversuch einen wichtigen Beitrag zur Suizidprävention leisten können. Die empirische Fundierung und Dissemination spezifischer suizidfokussierter Psychotherapie ist jedoch unzureichend und es bedarf strategischer und konzertierter Forschungsbemühungen, um hier Fortschritte zu erzielen.

## Footnotes

1 Es sei allerdings angemerkt, dass TAU alleine nicht selten einen deutlich positiven Effekt zeigt [24].
